# Hierarchical cluster analysis to identify the homogeneous desertification management units

**DOI:** 10.1371/journal.pone.0226355

**Published:** 2019-12-18

**Authors:** Farhad Zolfaghari, Hassan Khosravi, Alireza Shahriyari, Mitra Jabbari, Azam Abolhasani

**Affiliations:** 1 Higher Educational Complex of Saravan, Saravan, Sistan and Baluchestan, Iran; 2 Faculty of Natural Resources, University of Tehran, Tehran, Iran; 3 Faculty of Environmental Science, University of Sistan and Baluchestan, Zahedan, Sistan and Baluchestan, Iran; Hellenic Agricultural Organization - Demeter, GREECE

## Abstract

Since in most mapping models geometric mean of different criteria are used to determine the desertification intensity, one of the most important issues in desertification studies is understanding the similar areas, which require similar management after determining the desertification intensity map. Two similar classes of desertification intensity may require different management due to differences in the criteria that affect its desertification severity. Therefore, after determining the geomorphological facies as the working units in Sistan plain, we used hierarchical cluster analysis to identify the homogeneous environmental management units (HEMUs) based on indices of MEDALUS model. According to the MEDALUS model, the studied area was divided into two categories namely medium and high desertification classes. Working units (geomorphological facies) are classified into five clusters according to HEMUs analysis based on climate, soil, vegetation, and wind erosion criteria. The first cluster (C11) include six facies with moderate and severe desertification; in all of these units the main effective factor was wind erosion, so they need the same management decisions controlling wind erosion. Two working units (1 and 4) with the same desertification severity were placed in two different clusters due to the main factors affecting each other. The results of the Mann-Whitney test showed that the value of the test statistics was 79. Also, the value of Asymp.Sig was obtained to be 0.018, which is less than 0.025 (two-tailed test), and it can be concluded that the classification of work units in the two models, clustering and desertification, is not equal (P<0.05). So It seems that using cluster analysis to identify the same units, which need the same management decision after preparing the desertification intensity, is necessary.

## Introduction

A unique strategy for utilization of natural resources is commensurating with the physical power and the economic and social characteristics of the dominant region that is necessary to prevent land degradation and its geographic spread. In other words, protection, restoration, and reconstruction of natural areas will be possible when their natural and biological capacity is evaluated regarding restrictions [1,2]. Therefore, various zoning methods have been innovated and used from the past up to now as the methods evolved synchronously with the progress of science and technology development.

Ahmadi (1995) believed that the working unit could be considered as a study unit so that all studies and samples accomplished in that [3]. After facies were identified, he stated that the sequence could be concluded with the help of GIS and then working units are obtained. Then, maps of hydrology, soil, vegetation, and erosion can be used in a GIS environment to distinguish the characteristics of each work unit. Verstappen (1983) reported that geomorphological map is a valuable scientific tool and has two theoretical and practical functions [4]. The importance of geomorphological mapping is dependent on the recent progress and digital processes. Definition, description, and scientific value of the homogeneous unit’s map have attracted the researchers’ attention. Maps of landform units made applying geomorphology concepts elementary and understandable [5].

Managing the environmental regions in accordance with the sustainable regional development mandate is not an easy process since balancing the socioeconomic development and environmental conservation is often challenging [6]. This balance possibly will vary based on the high variability of the prime elements of the ecological system, i.e., the natural and socioeconomic subsystems [7].

The aim of the integrated environmental management is keeping the relationship between the resources of these two subsystems and their exploitation sustainable while avoiding (or alleviating) possible struggles and decreasing the uncertainties in terms of planning and decision-making [6]. Nevertheless, the appropriate management of a desert region requires a clear picture of the desertification model criteria in terms of each specific unit of territory. By sharing and accepting this vision, it would be possible to develop specific criteria to adapt uses of desert areas, settle possible conflicts, and ease the decision-making process.

It is possible to justify the spatially heterogeneous environment by selecting the homogeneous environmental management units (HEMUs), distinct homogeneous areas, or groups with comparable features (for similar approaches, See) [3,8,9]. Then, it would be necessary to link up these territorial units to a strategic national plan for operational management units [10]. These units lay the foundation for research and gathering data, and consequently, they will turn into the limits defining areas with similar land characteristics selected as decision criteria to plan and assessment [11]. Some analytical methods have been applied including multivariate classifications/clustering, factor analysis, fuzzy logic, multi-criteria analysis, and spatial overlapping [11–19].

Biophysical features like geomorphology, climate, vegetation, and biodiversity have been used to have the most comprehensive definitions of HEMU [6]. Nevertheless, developing an integrated vision of the desert zone requires the integration of the desertification model criteria into the process. It is possible to recognize groups having comparable environmental features by applying the clustering method. The analytical process that results in regionalization could be classified into separate approaches: hierarchical unit grouping and segmenting [19]. Typically, these two approaches elevate the regions according to a hierarchical criterion of higher scale unit; therefore, units are detectable as either belonging to a more upper area or forming one [15].

In most desertification studies, following the preparation of desertification intensity map and determination of desertification intensity classes in the study units, the management type is recommended based on the desertification intensity class [20–23]. In all these studies, management plans have been presented and developed following the preparation of desertification intensity maps based on common desertification models and determination of desertification risk classes in work units. In these studies, the same desertification intensity and desertification risk classes were the basis of the management plan, and the similarities between work units in terms of the indices studied have not been considered in any studies in the literature. This study is designed to answer the question whether work units with the same desertification class necessarily need to have the same management plan or not?

In this study, we propose using cluster analysis in different working units after determining the desertification intensity map to identify the units that require the same management decisions. Thus, we examined four desertification criteria (climate, soil, vegetation and wind erosion with different indices) based on the MEDALUS model in the dry east region of Iran, and then used cluster analysis to examine if the two different classes of desertification require the same management decision or not.

## Materials and methods

### Study area

**Fig 1:**
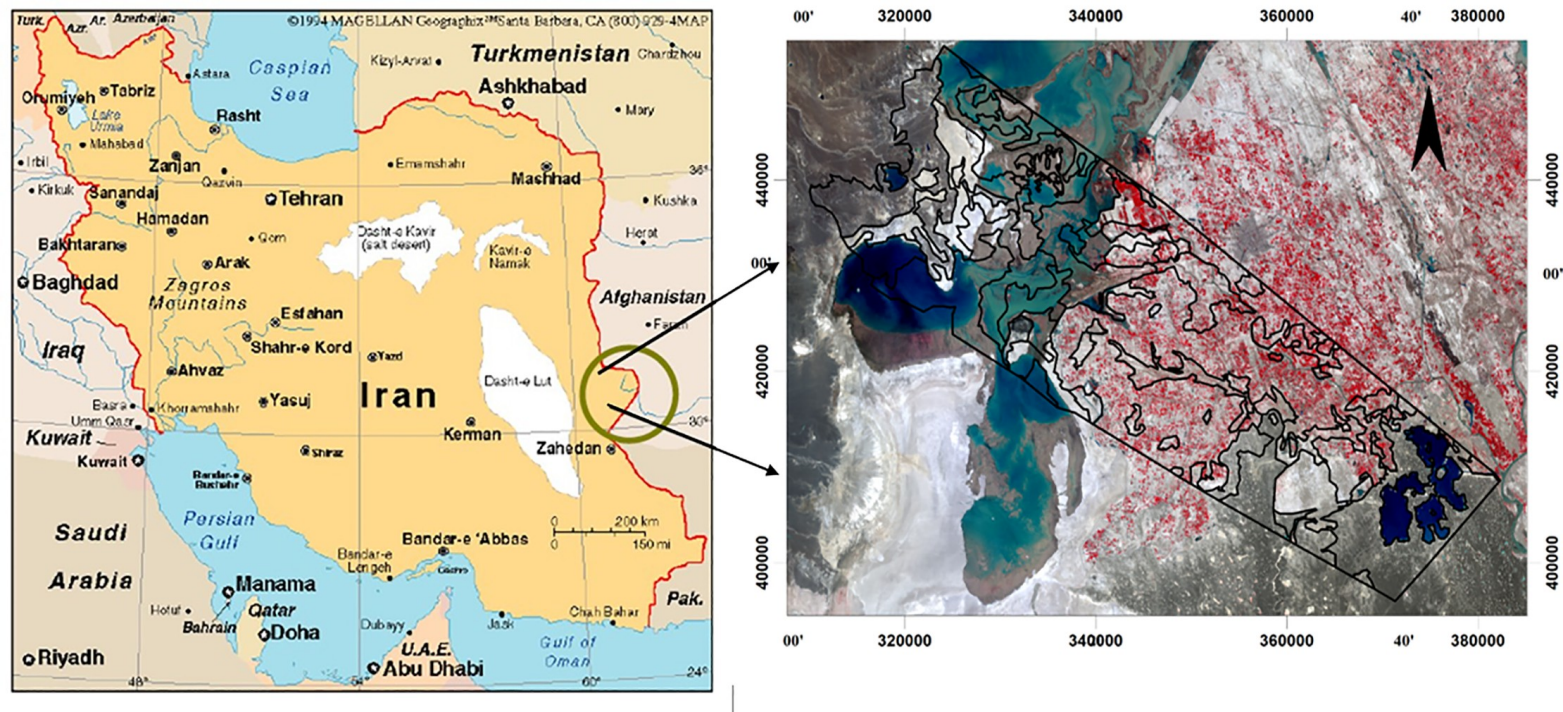
Study area[Image source from public domain: USGS EarthExplorer (https://earthexplorer.usgs.gov/)].

The Sistan plain, with an extension of about 15197 km^2^, is located in the Northeast of Sistan and Baluchistan Province (Iran) and at the end of the Hirmand River Basin and near the Afghanistan border (Fig 1).

This plain has specific geomorphological unevenness in spite of relative homogeneity. The climate of this region is xerothermic according to all of the climatic classifications. The geographical position of the study area is 21° 20΄ Northern latitudes and 61° 29΄ of Eastern longitudes. The average altitude of the plain is 490m. The average annual rainfall is 61.4 mm/year while the average annual temperature is 21°^C^ [24]. The percentage of relative humidity is 38 percent [25]. Potential evaporation and transpiration are between 4196 mm and 5700 mm [26]. The most rainfall was concentrated in fall and winter seasons (more than that indicated in the present study), and species were classified according to their growth habit. According to Domarten climate classification method, this region has desert climate [27].

### Hierarchical cluster analysis

Cluster analysis is a significant technique for classifying a ‘mountain’ of information into manageable, meaningful piles. It is a data reduction tool that creates subgroups that are more manageable than individual datum. It examines the full complement of inter-relationship between variables. In cluster analysis, it is not known which elements fit into which clusters. The data is reviewed to define the grouping or clusters.

Cluster analysis, like factor analysis, makes no distinction between dependent and independent variables. The entire set of interdependent relationships is examined. Cluster analysis is the obverse of the factor analysis. Factor analysis reduces the number of variables by grouping them into a smaller set of factors, but cluster analysis reduces the number of observations or cases by consolidating them into a smaller set of clusters. Hierarchical cluster analysis is the major statistical method for finding homogeneous groups of cases based on the measured characteristics [28]. It starts with each case as a separate cluster, i.e., there are as many clusters as cases, and then combines the clusters sequentially, reducing the number of clusters at each step until only one cluster is left. The clustering method uses the dissimilarities or distances between objects when forming the clusters. The SPSS software calculates ‘distances’ between data points regarding the specified variables.

A summary of the clustering as a general process is as follows:

- The distance is calculated between all initial clusters. In most analyses, individual cases will build up the initial clusters.
- Then, the distances are calculated again following the fusion of the two most similar clusters.
- Step 2 is done over repeatedly until all cases ultimately turn into one cluster.

Distance can be measured in a variety of ways [28].

There are some distance measures within SPSS. The squared Euclidean distance has been applied most frequently. The Euclidean distance between two values is the arithmetic difference [28].

The squared Euclidean distance is applied more frequently than the simple Euclidean distance to impose gradually greater weight on objects that are further apart. To determine how distance is measured, it is necessary to select the clustering algorithm, namely, the rules governing which points distances are determined to specify cluster membership [28,29].

Many methods have been introduced. SPSS has five clustering algorithms; Ward’s method is the most frequently used algorithms, which differs from other methods because of applying an analysis of variance approach to assess the inter-clusters distances. Generally, this method is very effective. The total sum of squared deviations from the mean of a cluster is computed to evaluate cluster membership. The criterion for fusion is yielding the minimum likely increase in the error sum of squares.

Euclidean distance coefficient specifies the distance between units; the greater distance implies making diverse managerial decisions. This method can be advantageous especially where the region has been a large extent and divided into various geomorphological units.

Instead of spending a lot of time and money for applying desertification strategies in single units, similar approaches have been used for units in a cluster; so, more reasonable results have been achieved. Some researchers used geomorphology levels to get the particular lines of distinction between areas [30–34]. Grouping similar or very different areas was their studies result.

In this study We used Agglomerative hierarchical clustering. This is a "bottom-up" approach, each observation starts in its own cluster, and pairs of clusters are merged as one moves up the hierarchy. This method builds the hierarchy from the individual elements by progressively merging clusters [28]. The first step is to determine which elements to merge in a cluster. Usually, we want to take the two closest elements, according to the chosen distance. To do that, we need to take the distance between elements and therefore define the distance between two clusters [29]. We used Ward's method (The increase in variance for the cluster being merged) to estimate the distance between two clusters. Ward's minimum variance method is a special case of the objective function approach originally presented by Joe H. Ward [35]. Ward suggested a general agglomerative hierarchical clustering procedure, where the criterion for choosing the pair of clusters to merge at each step is based on the optimal value of an objective function [29].

## Methodology

Major indices of desertification were determined based on the IMDPA (Iranian Model of Desertification Potential Assessment) and assessment of desertification intensity classified according to the MEDALUS model [36]. The MEDALUS model is one of the world's desertification assessment methods introduced by the European Commission in 1999 for desertification and land degradation studies. Higher accuracy is one of the important advantages of this method compared with other desertification assessment models. In this model, four indicators of soil quality, climate quality, vegetation quality and management are considered for desertification mapping. Each criterion has different indices. Each index gets a weight of 1 to 2, depending on its impact on desertification, according to experts and ground truth. Finally, each criterion's score is derived from the geometric mean weight of its indices. The desertification intensity (severity) map is also calculated from the geometric mean of the criteria and classified into four classes from low to very severe [37–38].

In this research, four indicators including soil, wind erosion, climate, and vegetation were selected based on the instructions of the MEDALUS model and according to the native conditions of the area presented under the IMDPA model. The weight of each indicator was calculated based on its indices. The procedure is that each indicator should be examined within the work unit.

IMDPA is a comprehensive desertification model that was developed by the University of Tehran to study the land degradation in arid and semi-arid regions of Iran [39]. Many studies have been done using this model in Iran [37–45]. The work units are determined according to slope, geology and land-use maps; however, since the slope was less than 2% in the study area, the geomorphological facies map was used to determine the work units. In this study, geobiofacies work units were considered. These work units are one of the environmental planning units, in which vegetation characteristics and land use type are considered in addition to the geomorphology of the area.

Desertification in each geomorphological facies was assessed using geographic information system (GIS) and MEDALUS model. In work units, all indicators were evaluated and the layer for each criterion was specified by calculating the geometric mean (Eq 1).

$$X={\left[\left(layer_{1}\right)\times \left(layer_{2}\right)\times \ldots \times \left(layer_{n}\right)\right]}^{1/n}$$ (1)

X: the desired criterion

Layer: Indicators of each criterion

N: Number of indicators for each criterion

To achieve the desired goal in this study, which is determining the homogeneous environmental management units (HEMUs) of the area, we did the following steps that are briefly described.

**Fig 2:**
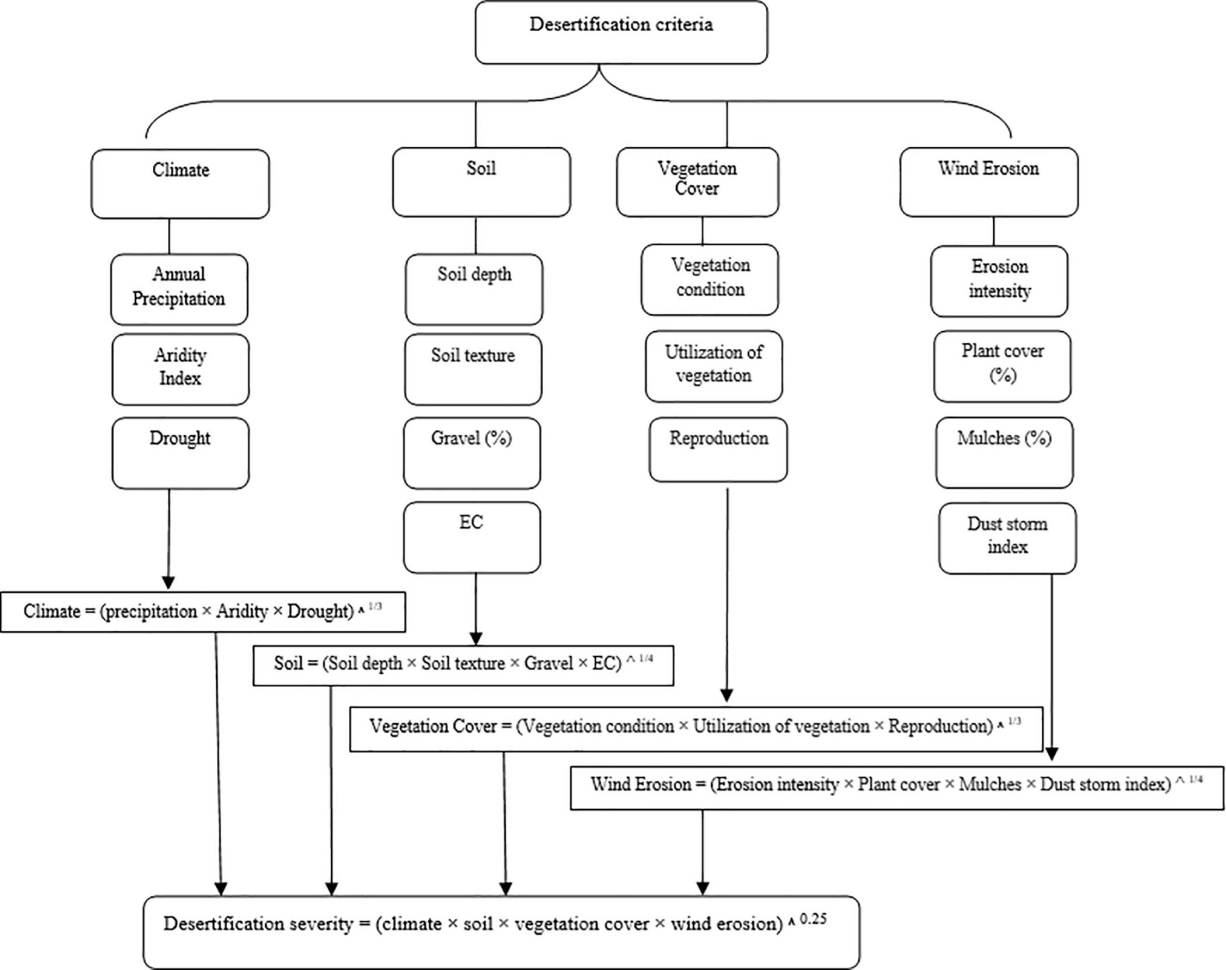
Flowchart and different steps of determining the intensity of desertification based on IMDPA model in each working unit.

- Positioning the region and existing phenomena such as geomorphology facies in the area that were determined using the topographic maps (1:50000 and 1:250000) and Landsat satellite images (TM with the requirement combined bands) and matching them with the report of desertification and combating wind erosion studies in Sistan region [46] and field visits.
- Collecting data and information on climatology, geomorphology, vegetation and soil issues based on the determined work units.
- Identifying and determining the dominant processes of land degradation through the field visits according to the sub-criteria and criteria derived from the analysis of MEDALUS procedures associated with the criteria and sub-factors consistent with the study area in each working units (Fig 2).
- Determining the appropriate criteria and sub-factors for the evaluation of the land degradation process in each studied working units. Tables 1 to 4 show all indicators in this study.

**Table 1:** Indicators of climate criterion.

Range Of Score	Annual Precipitation (mm)	Aridity Index (WD^a^)	Drought Period (Year)
Low (1–1.24)	280<	150–180	3–4
Medium (1.25–1.49)	150–280	150–120	5–6
High (1.5–1.74)	75–150	120–90	6–7
Very High (1.75–2)	<75	0–90	>7

^a^: Wet Days

**Table 2:** Indicators of soil criterion.

Range Of Score	Soil depth (cm)	Soil texture	Electrical conductivity(dsm^-1^)	Gravel (%)
Low (1–1.24)	80<	Clay, clay loam	<5	<15
Medium (1.25–1.49)	50–80	Fine loam	5–8	15–35
High (1.5–1.74)	20–50	Coarse loam	9–16	35–75
Very High (1.75–2)	<20	Sand, sandy loam	>16	>75

**Table 3:** Indicators of vegetation cover criterion.

Range Of Score	Vegetation condition	Utilization of vegetation cover	Reproduction
Low (1–1.24)	Invader species are<5% of vegetation cover and annual plants >25%, Surface litter is >90%, Foliage cover of perennials is >85%	cutting of brush and uproot of the shrub is not seen, Stocking rate is equal to the rang capacity	Reproduction of plants are done naturally, The region does need not to reclamation projects, Decreases and increaser species are 70 and 30% respectively, and invaders ones are not seen
Medium (1.25–1.49)	Invader species are5-20% of vegetation cover annual plants 25–50%, Surface litter is70-90%, Foliage cover of perennials is 15–30%	trees are more than annual biomass, Stocking rate is a little more than annual production	Reproduction of plants are access able with low expense, Range improvement projects are successfull and effective, Decreases and increaser species are dominant, and invaders ones are seen seldom
High (1.5–1.74)	Invader species are20-50% of vegetation cover and annual plants are dominant, Surface litter is 30–70%, Foliage cover of perennials is 5–15%	cutting of brush, bush, and trees are apparent, Grazing is more than capacity	Regeneration of plants involve high expense, Range improvement plans are a success to some extent, Invaders and increaser species are dominant and decrease ones are not seen
Very High (1.75–2)	Invader species are>50% of vegetation cover, and annual plants are dominant, Surface litter is <30% foliage cover of perennials is <5%	Heavy cutting of brush, bush, and trees, Heavy stocking rate	Regeneration of plants are impossible (ecological problem), Range improvement projects have not successes till now, Invaders species are dominant, and increaser ones are not seen

**Table 4:** Indicators of wind erosion criterion.

Range of Score	Erosion intensity	Plant cover percentage	Mulches percent(gravel >2mm)	Dust storm index(days)
Low (1–1.24)	80<	PC>40	MC>80	<10
Medium (1.25–1.49)	50–80	20<PC<40	40<MC<80	10–30
High (1.5–1.74)	20–50	10<PC<20	20<MC<40	30–60
Very High (1.75–2)	<20	<10	MC<20	>60

**Fig 3:**
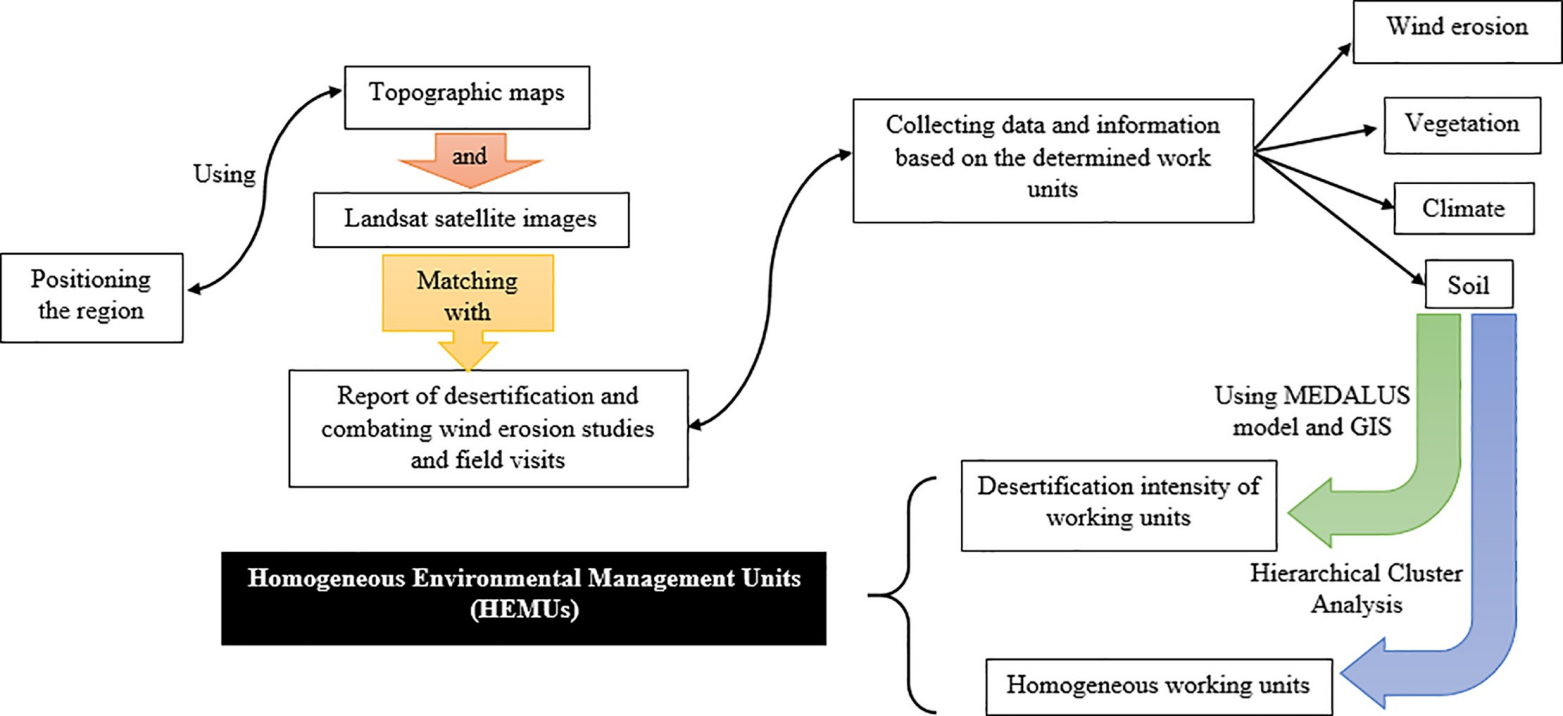
Graphical abstract of the methodology.

- To determine the homogeneous environmental management units (HEMUs) of the area, we carried out a hierarchical cluster analysis by the use of Ward’s Minimum Variance method and applying Squared Euclidean Distance as the distance or similarity measure. It is effective in calculating the optimum number of clusters.
- The data were analyzed and compared using SPSS_23_. The Graphical abstract of the methodology is shown in Fig 3.

## Results

**Fig 4:**
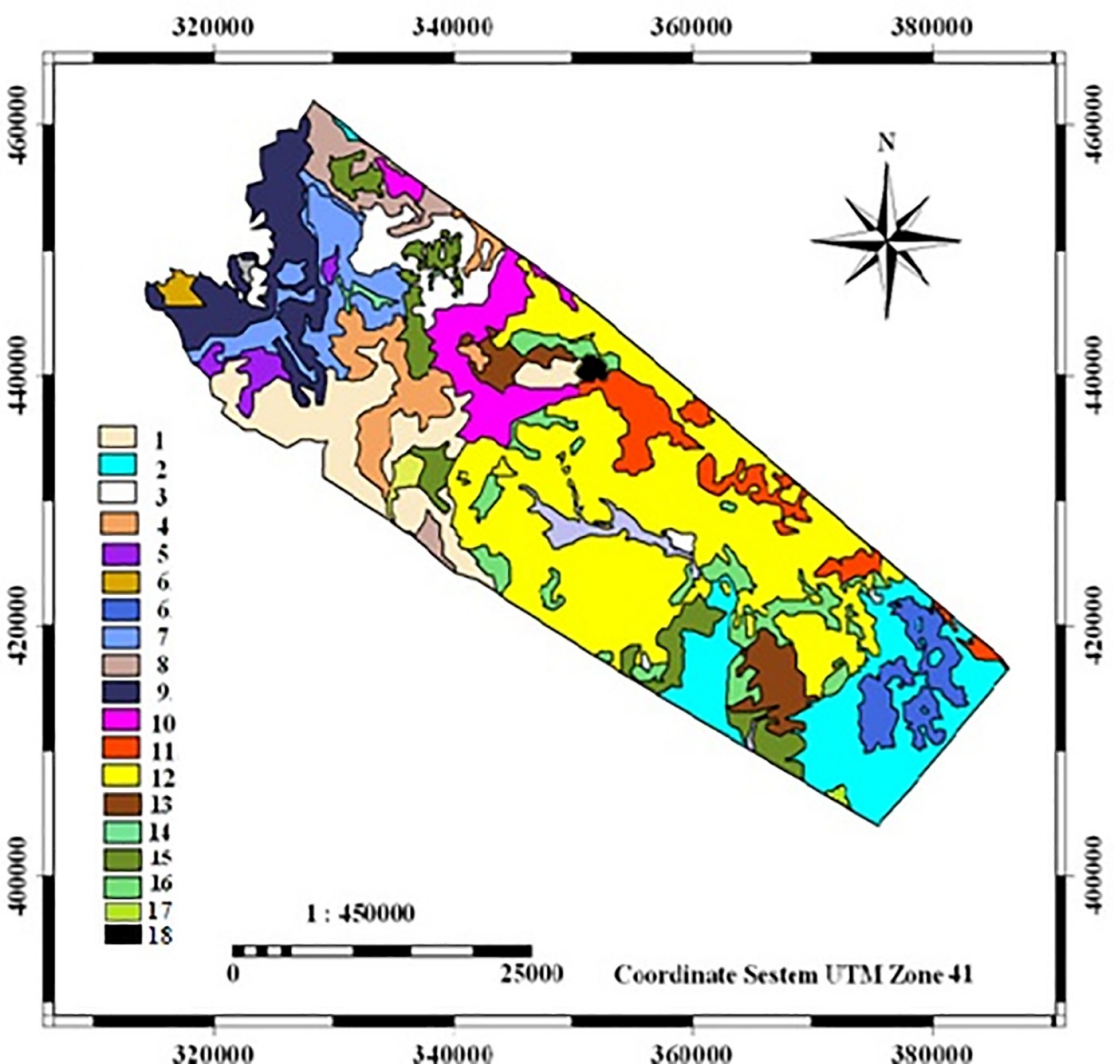
Location of geomorphological facies in the area.

In this research geomorphological units were identified according to the basic maps of the region and in each group, four main criteria that were effective in desertification in the study area were checked out and classified with cluster analysis. The distribution of geomorphological facies in the study area, code and the names of geomorphological facies are shown in Fig 4 and Table 5, respectively.

**Table 5:** Code and names of geomorphological facies in the area.

Facies Code	Facies Name
1	Playa fans with fine sediment
2	Eroded marl terrace with low vein combined with claypan and hole basin
3	Relatively stiff silty clay lands
4	Relatively hard clay zone with low halophyte vegetation
5	Salina
6	Salt and bloated lands
7	Gravelly plain with large vein and high density combined with the ups and downs
8	Salt and bloated lands combined with a hard clay pan
10	Gravelly plain with medium vein and high density combined with clay pans
11	Dry and hard shell with Tamarix
12	Abandoned lands and sandy agricultural lands
13	Agricultural lands (mainly abandoned in drought)
14	Sand dunes and zone
15	Medium gravelly plain and low to medium density
16	Sand dunes
17	Hard clay lands with low Tamarix and halophyte vegetation

**Fig 5:**
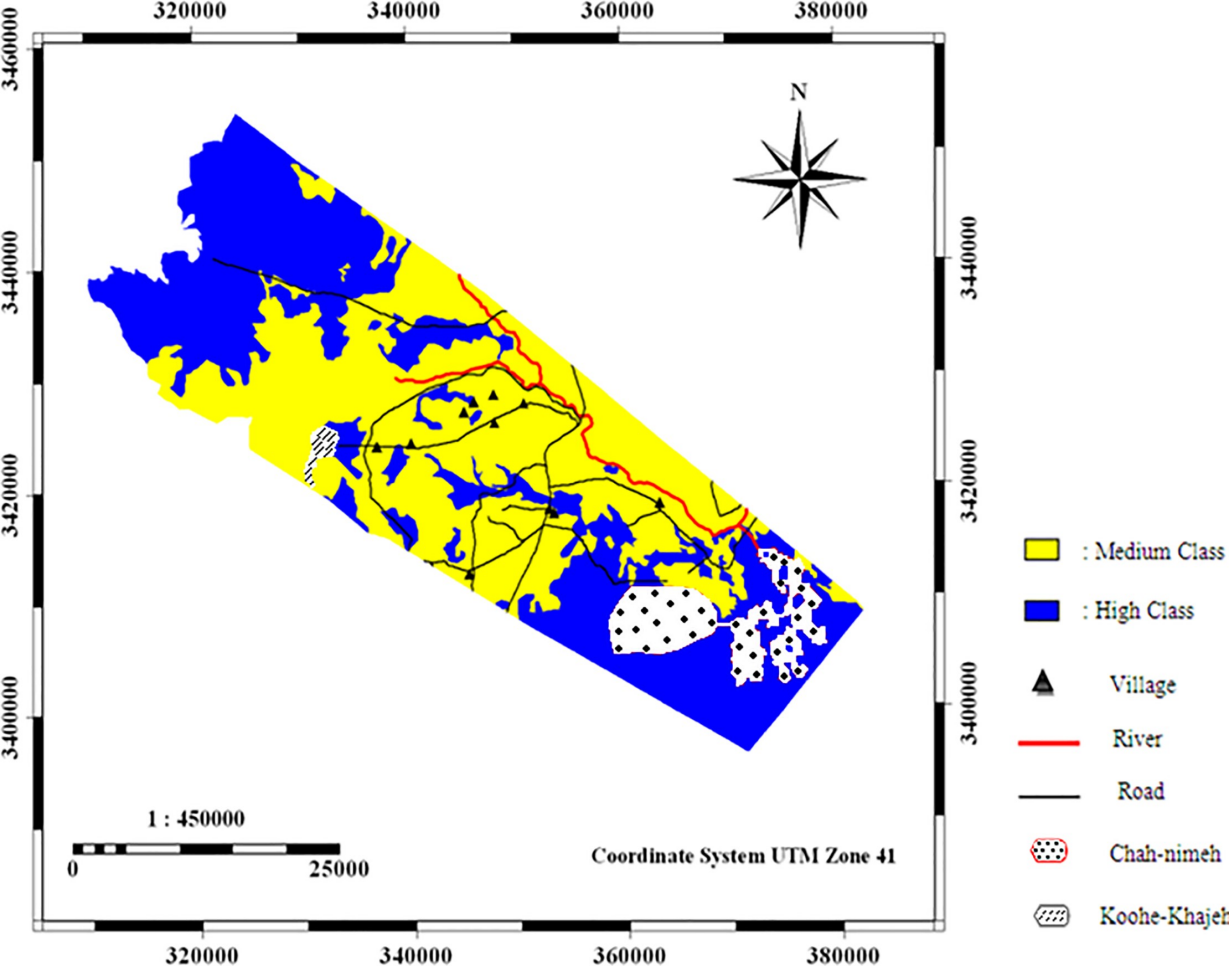
Map of current desertification severity caused by climate, soil, vegetation and wind erosion.

As shown in Tables 1 to 4, the current desertification status was mapped according to four criteria including climate, soil, vegetation, wind erosion, and the final scores. The results of desertification map are shown in Table 6 and Fig 5.

**Table 6:** The geometric mean of the quantitative values of each criterion on geomorphological facies and its cluster group.

Facies code	Geometric mean of each criteria				Desertification severity	Cluster number
	climate	soil	wind erosion	vegetation		
1	1.57	1.32	1.53	1.55	Medium	C2
2	1.57	1.52	1.69	1.55	high	C1222
3	1.57	1.34	1.75	1.54	high	C1221
4	1.57	1.3	1.69	1.45	Medium	C11
5	1.57	1.52	1.83	1.52	high	C2
6	1.57	1.44	1.49	1.49	Medium	C11
7	1.57	1.32	1.75	1.49	high	C2
8	1.57	1.3	1.78	1.49	high	C11
9	1.57	1.45	1.71	1.52	high	C11
10	1.57	1.30	1.48	1.52	Medium	C1221
11	1.57	1.30	1.48	1.52	Medium	C2
12	1.57	1.42	1.66	1.52	high	C121
13	1.57	1.28	1.65	1.48	Medium	C121
14	1.57	1.51	1.75	1.52	high	C1221
15	1.57	1.37	1.52	1.61	high	C2
16	1.57	1.38	1.7	1.64	high	C11
17	1.57	1.51	1.74	1.52	high	C1221

**Fig 6:**
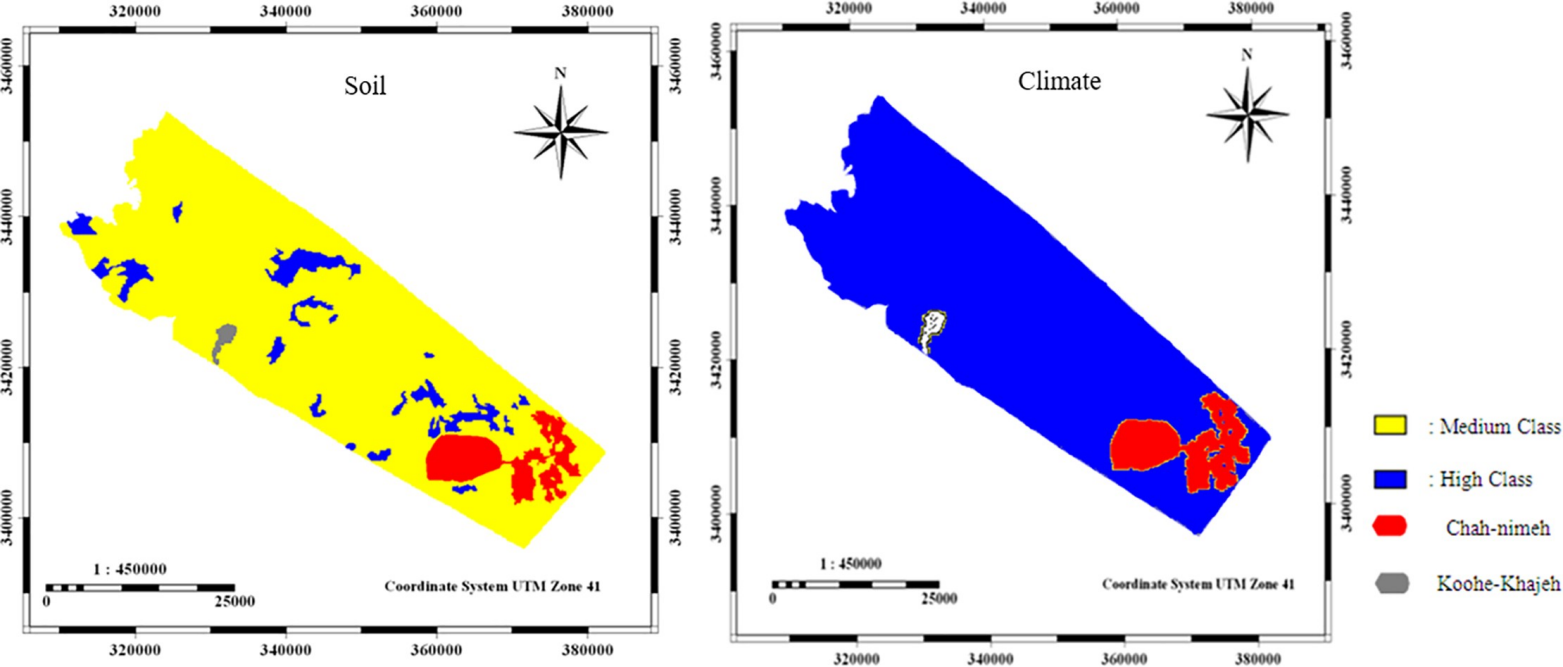
Maps of soil and climate layer criteria.

The soil and climatic criteria with an average score of 1/36 and 1/57, respectively, were categorized in moderate and severe classes of desertification (Fig 6).

**Fig 7:**
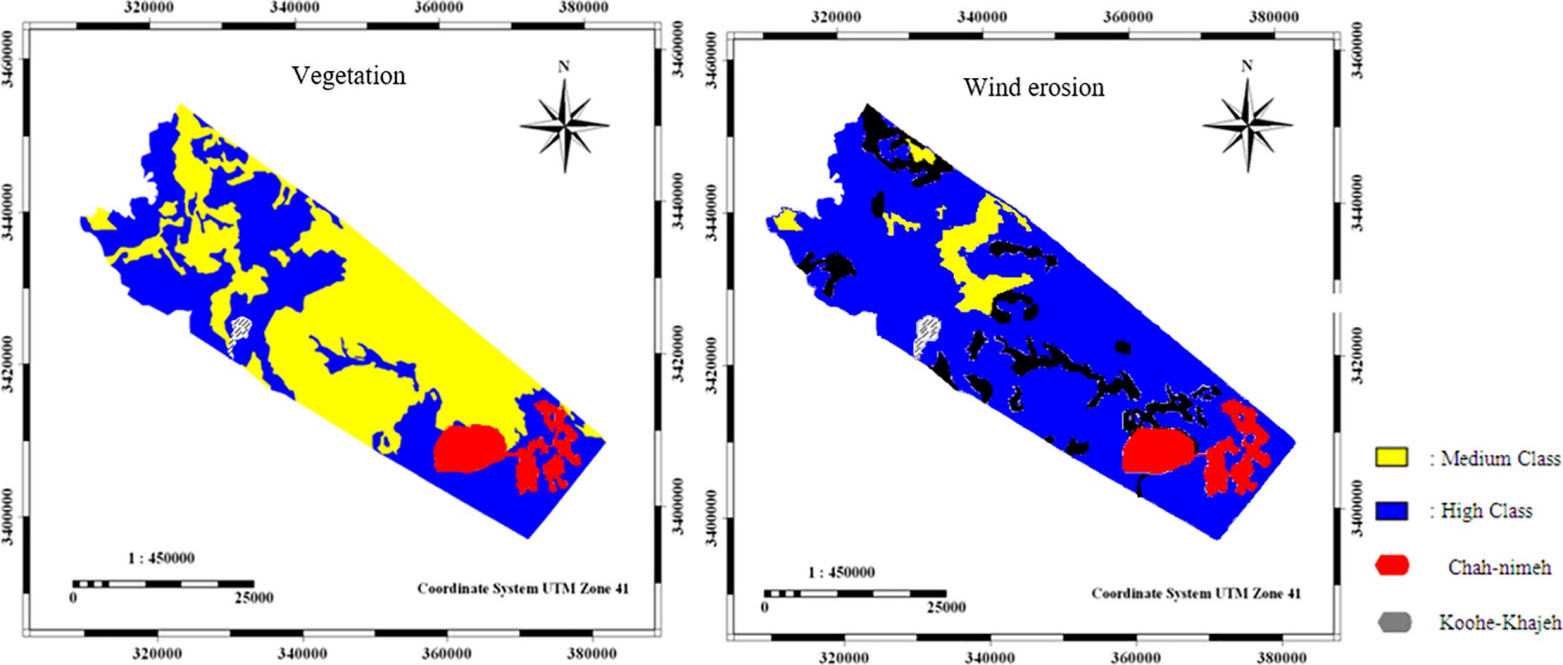
Maps of vegetation, and wind erosion layer criteria.

The vegetation and wind erosion criteria had an average score of 1/51 and 1/67, respectively, which were categorized in high class of desertification (Fig 7).

**Fig 8:**
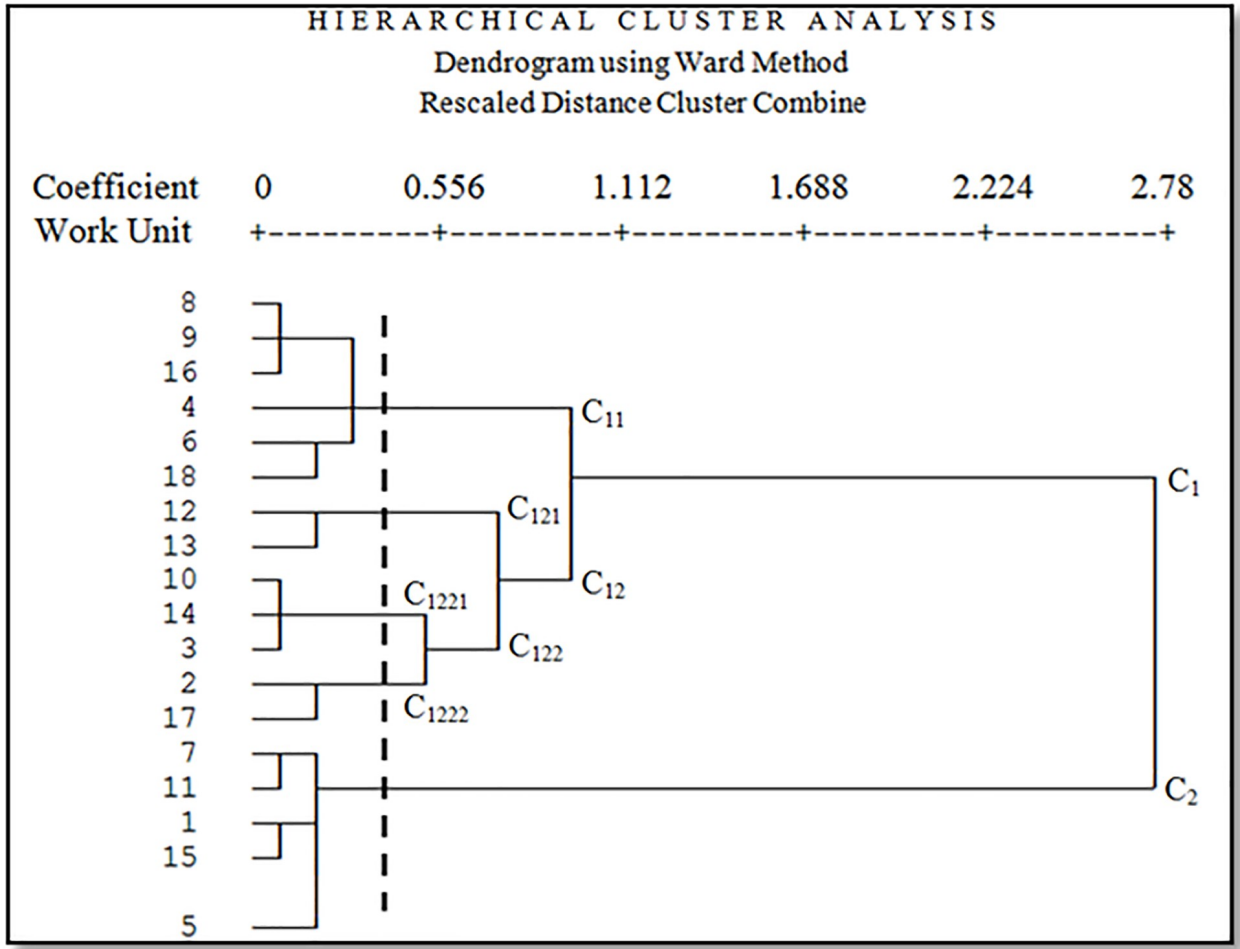
Dendrogram of all working units based on four criteria climate, soil, vegetation, and wind erosion.

The results showed that geomorphological units were classified into five clusters based on four main criteria including climate, soil, vegetation, and wind erosion. Fig 8 shows the dendrogram of all geomorphological units based on four criteria including climate, soil, vegetation, and wind erosion. The horizontal axis represents the Euclidean Distance between units.

**Fig 9:**
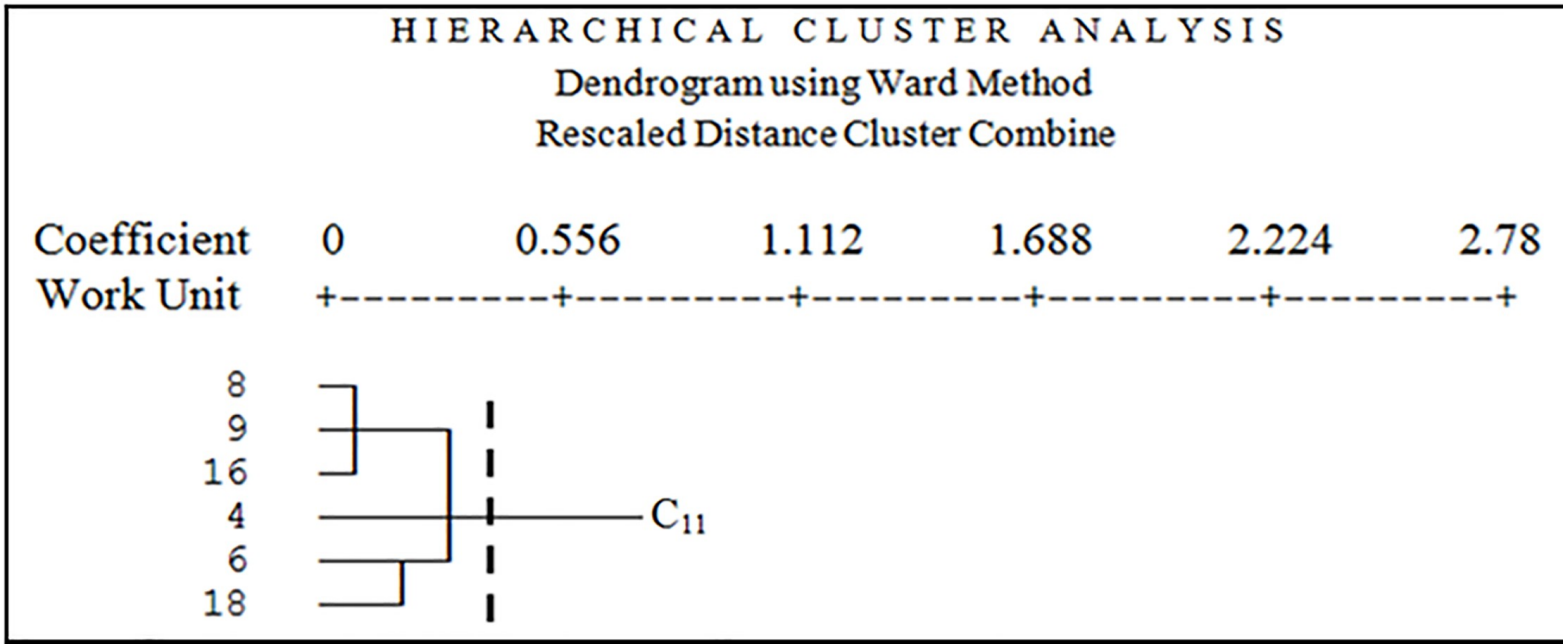
Dendrogram of the first cluster.

In Fig 8, all units are classified in C_11_, C_121_, C_1221_, C_1222_ and C_2_ clusters (the cutting line is from where there is a maximum distance between groups) based on analyzed criteria. Cutting line was chosen lower than the average Euclidean Distance (0.1853). C_11_ cluster contains six geomorphological units; C_121_ cluster contains two geomorphological units; C_1221_ cluster contains three geomorphological units; C_1222_ cluster contains two geomorphological units, and C_2_ cluster contains five geomorphological units. Fig 9 shows cluster C_11_ geomorphological facies (4, 6, 8, 9, 16, and 18).

**Fig 10:**
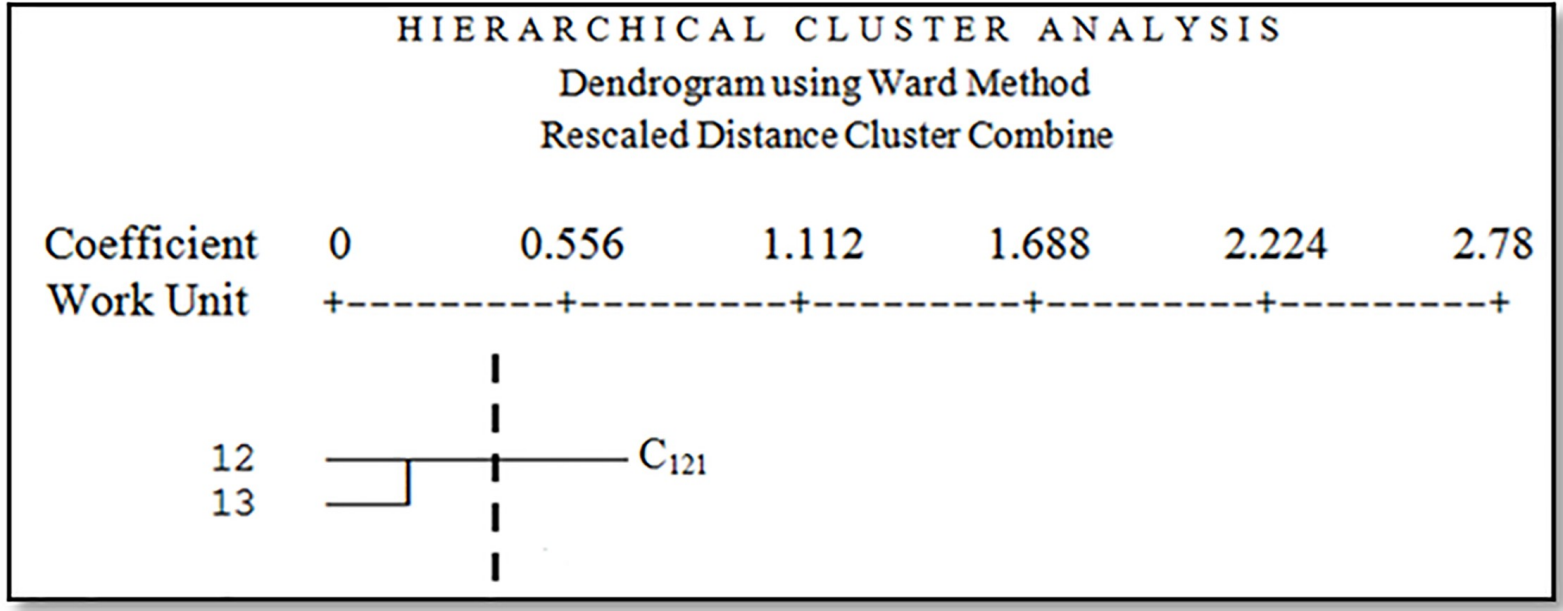
Dendrogram of the second cluster.

Facies 4 is the relatively hard clay zone with low halophyte vegetation. The dominant vegetation type in this facies is *Tamarix aphylla* with 32 percent canopy. Other Species with this vegetation type are *Salsola canescens*, *Suaeda fruticosa*, *Aeluropus littoralis*, *Calligonum sp*. and *Ephedra Strobilaceum*. Facies 6 is Salt and bloated lands. The dominant vegetation type in this facies is *Tamarix aphylla*. Facies 8 is Salt and bloated lands combined with hard clay pan. The overriding vegetation type in this facies is degraded *Aeluropus littoralis* and species like *Tamarix aphylla*, *Haloxylon persicum*, and *Alhagi camelorum* have been observed too. Facies 9 is a dry and hard shell without vegetation or with shallow vegetation. The dominant vegetation types in this facies are *Tamarix aphylla* and *Alhagi camelorum* with 17 percent canopy. You can also see *Salsola canescens*, *Suaeda fruticosa* in this facies. Facies 16 is mainly sand dunes. The dominant vegetation types in this facies are *Tamarix aphylla* and *Alhagi camelorum*. Facies 18 is abandoned salt and bloated lands sometimes with Nebka. The dominant vegetation types in this facies are *Tamarix aphylla*, *Haloxylon persicum*, and *Seidlitzia rosmarinus*. The second cluster (C_121_) included two working units, facies 12 and 13 (Fig 10).

**Fig 11:**
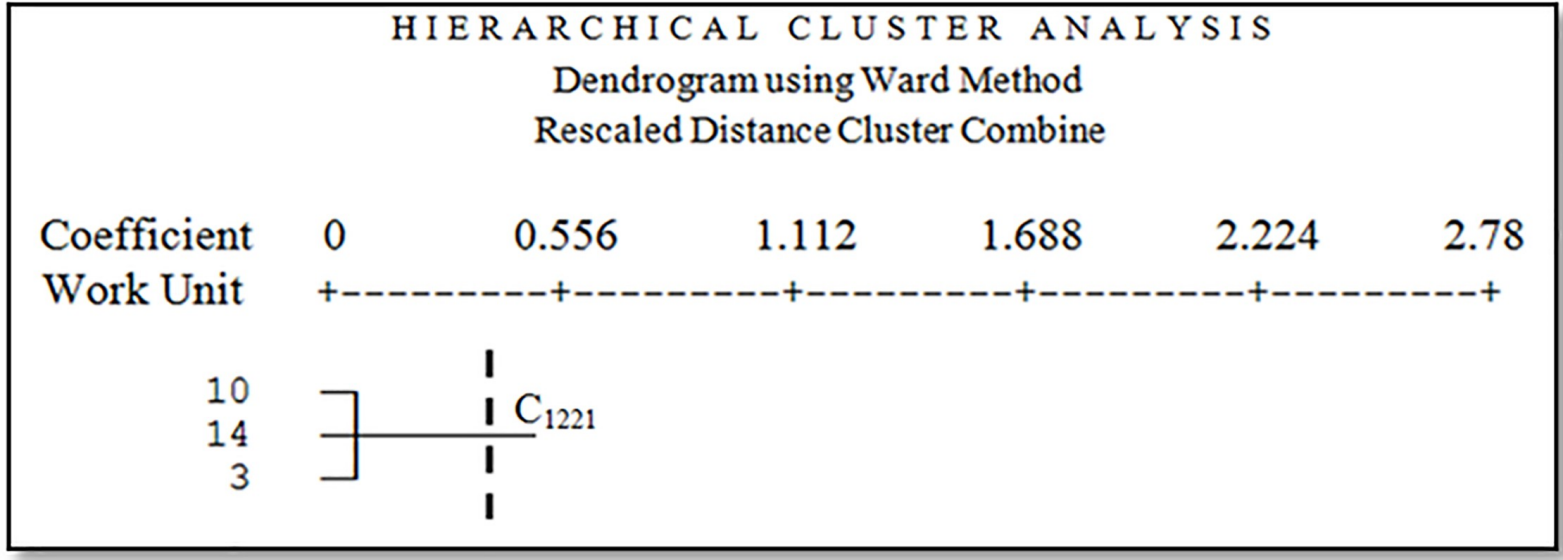
Dendrogram of the third cluster.

Facies 12 is abandoned lands and sandy agricultural lands. The dominant vegetation types in this facies are *Tamarix aphylla*, *Salsola canescens* and *Alhagi camelorum* with 20 percent canopy. Facies 13 is agricultural lands that mainly abandoned in drought. The dominant vegetation types in this facies are *Alhagi camelorum*, *Tamarix aphylla*, and *Prosopis sp*. The third cluster (C_1221_) included three working units, facies 3, 10 and 14 (Fig 11).

**Fig 12:**
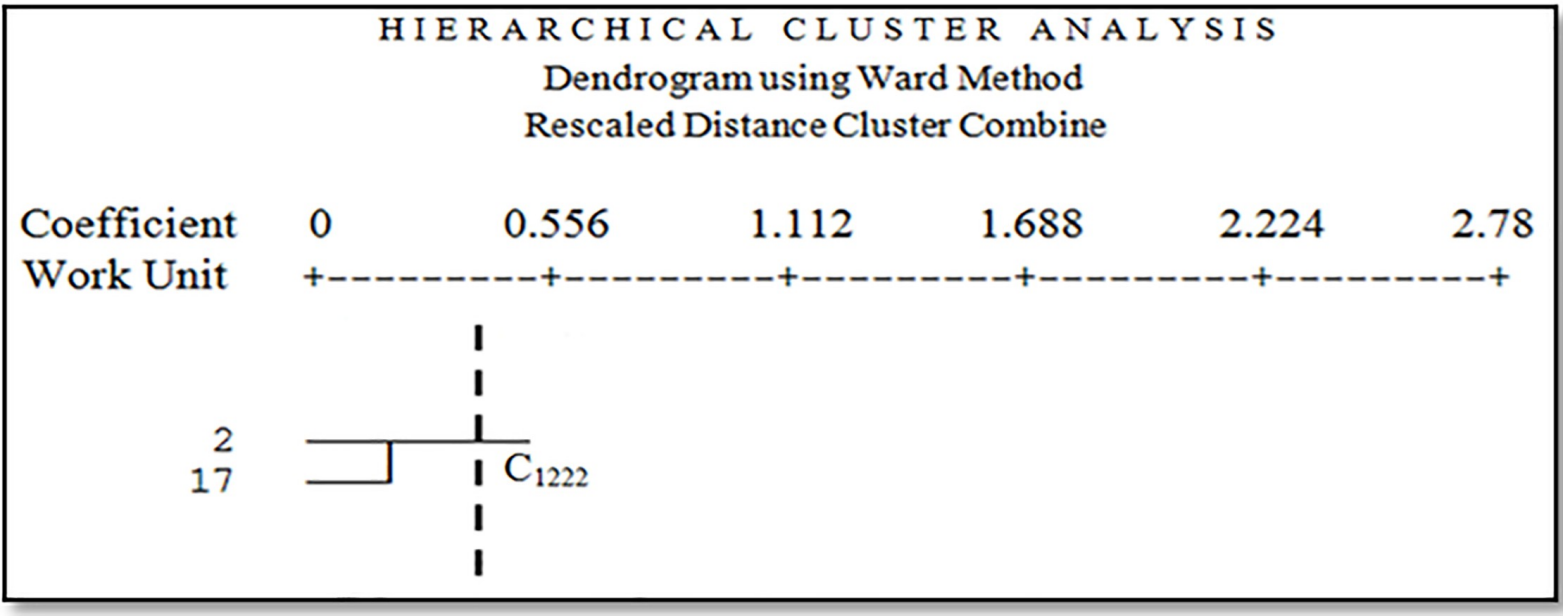
Dendrogram of the fourth cluster.

Facies 3 is relatively stiff silty clay lands. The dominant vegetation types in this facies are *Salsola canescens*, *Prosopis sp*. and *Cyperus sp*. with 35 percent canopy. Species with this vegetation types are *Tamarix aphylla*, *Alhagi camelorum*, and *Aeluropus littoralis*. Facies 10 is gravelly plain with medium vein and high density combined with clay pans. The dominant vegetation types in this facies are *Ephedra strobilaceum* and *Zygophyllum eurypterum* with 11 percent canopy. Species with this vegetation types are *Aeluropus littoralis*, *Tamarix aphylla*, *Alhagi camelorum*, *Haloxylon persicum*, and *Artemisia scoparia*. Facies 14 is sand dunes and zone. The dominant vegetation types in this facies are *Tamarix aphylla*, *Cyperus sp*. *Alhagi camelorum* with 35 percent canopy. Species with this vegetation types are *Prosopis sp*. and *Salsola canescens*. The fourth cluster (C_1222_) included facies 2 and 17 (Fig 12).

Facies 2 is eroded marl terrace and low vein combined with claypan and basin hole. The dominant vegetation types in this facies are *Tamarix aphylla*, *Alhagi camelorum* and *Salsola canescens* with 12 percent canopy, and also you can see *Aeluropus littoralis* in this facies.

**Fig 13:**
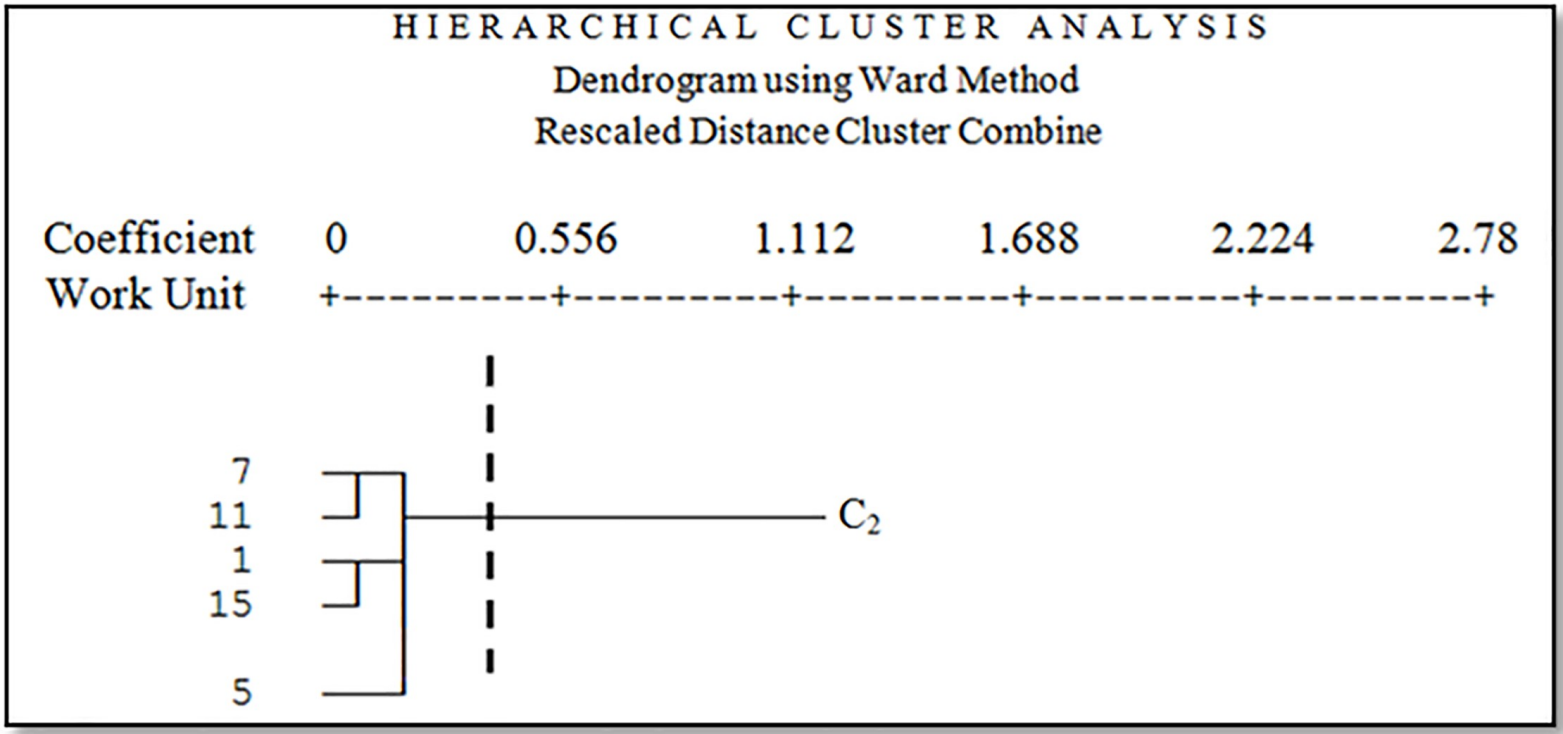
Dendrogram of the fifth cluster.

Facies 17 is hard clay lands with low Tamarix and halophyte vegetation. The dominant vegetation types in this facies are *Tamarix aphylla* and *Aeluropus littoralis* with 28 percent canopy, and you can see *Haloxylon persicum*, *Ephedra strobilaceum*, *Seidlitzia rosmarinus*, and *Calligonum sp*. The fifth cluster (C_2_) included facies 1, 5, 7, 11 and 15 (Fig 13).

Facies 1 is playa fans with fine sediment. The dominant vegetation types in this facies are *Tamarix aphylla* and *Aeluropus littoralis* with 12 percent canopy, and you can see *Ephedra strobilaceum*, *Calligonum sp*. and *Artemisia scoparia*. Facies 5 is Salina. The dominant vegetation type in this facies is *Tamarix aphylla* with 30 percent canopy, and you can see *Haloxylon persicum* and *Salsola canescens*. Facies 7 is gravelly plain with large vein and high density combined with the ups and downs. The dominant vegetation type in this facies is *Tamarix aphylla* with 45 percent canopy, and you can see *Salsola canescens*, *Suaeda fruticosa*, *Haloxylon persicum*, and *Zygophyllum eurypterum*. Facies 11 is a dry and hard shell with Tamarix. The dominant vegetation types in this facies are *Tamarix aphylla* and *Aeluropus littoralis*, and you can see *Alhagi camelorum*, *Salsola canescens*, *Haloxylon persicum* and *Atriplex canisens*. Facies 15 is medium gravelly plain and low to medium density. The dominant vegetation types in this facies are *Tamarix aphylla* and *Suaeda fruticosa* with 35 percent canopy.

## Discussion

As shown in the cluster analysis, working units in each cluster were similar in some characteristics such as soil and vegetation type, but not equal. Webster and Oliver, (1990) confirmed the performance of cluster analysis on test and differentiation of geomorphological units and soil specifications in Brazil [47]. Working units in adjacent clusters are more similar than working units in further clusters. Multivariate analysis can specify minor differences within a group to major differences between groups [48]. According to Adam et al., (1992) in soil studies, cluster analysis is very suitable to organize the degree of similarity, so it is used to achieve classification goals [49].

Cluster analysis in geomorphological studies shows further details of the relationship between soil properties that cannot be estimated at the ground level [50]. Milton et al., (2012) analyzed physicochemical properties of the soil in three regions with multivariate analysis and confirmed the results of cluster analysis of the regions based on the first pattern [51].

In a first cluster, there are six facies that all of them have weak halophyte vegetation and the dominant vegetation type in these facies is *Tamarix aphylla*, *Suaeda fruticosa*, *Haloxylon persicum*, and *Alhagi camelorum*. All of these facies include clay pan, dry shell and salt and bloated lands due to their geomorphology. These facies have the same conditions in the process of planning for implementation of desertification due to the mentioned similarities. In this cluster, although the facies 8, 9, 16, 18, and the facies 4 and 6 are classified in the high and medium class of desertification, they are placed in the similar cluster because these facies need the same management for wind erosion and vegetation cover. Therefore, to reduce the risk of desertification in this cluster (C11), the pebble mulch and spreading of vegetation residues are used on the surface to prevent the deterioration of surface erosion and maintenance of surface soil is the best and appropriate function.

In the second cluster (C121), there are two facies, both of them include agricultural lands abandoned in drought, the dominant vegetation types are *Prosopis sp*., *Alhagi camelorum* and *Tamarix aphylla*. In this cluster, the best management proposal is using biological windbreak and prevent livestock entry.

Three facies were classified in the third cluster. These facies include relatively stiff clay lands and gravelly plain combined with clay pan and sand areas. The dominant vegetation types in these facies are *Zygophyllum eurypterum*, *Ephedra strobilaceum*, and *Cyperus sp*. with about 30 percent canopy. Due to the high sensitivity of surface soil in all homogeneous units located in C1221 cluster, we suggest using methods to increase the adhesion of aggregate particles such as clay mulch.

In the fourth cluster (C1222), there are two facies including eroded marl terrace combined with hard clay pan. In these facies, you can see low vegetation of *Salsola canescens*, *Tamarix aphylla*, *Aeluropus littoralis*, *Calligonum sp*. and *Seidlitzia rosmarinus*. In this cluster, which includes two work units with a low percentage of some delicate species, the management plan should be focused on restoring vegetation and using the exclusion of the area to give sufficient opportunity for plant regeneration.

**Fig 14:**
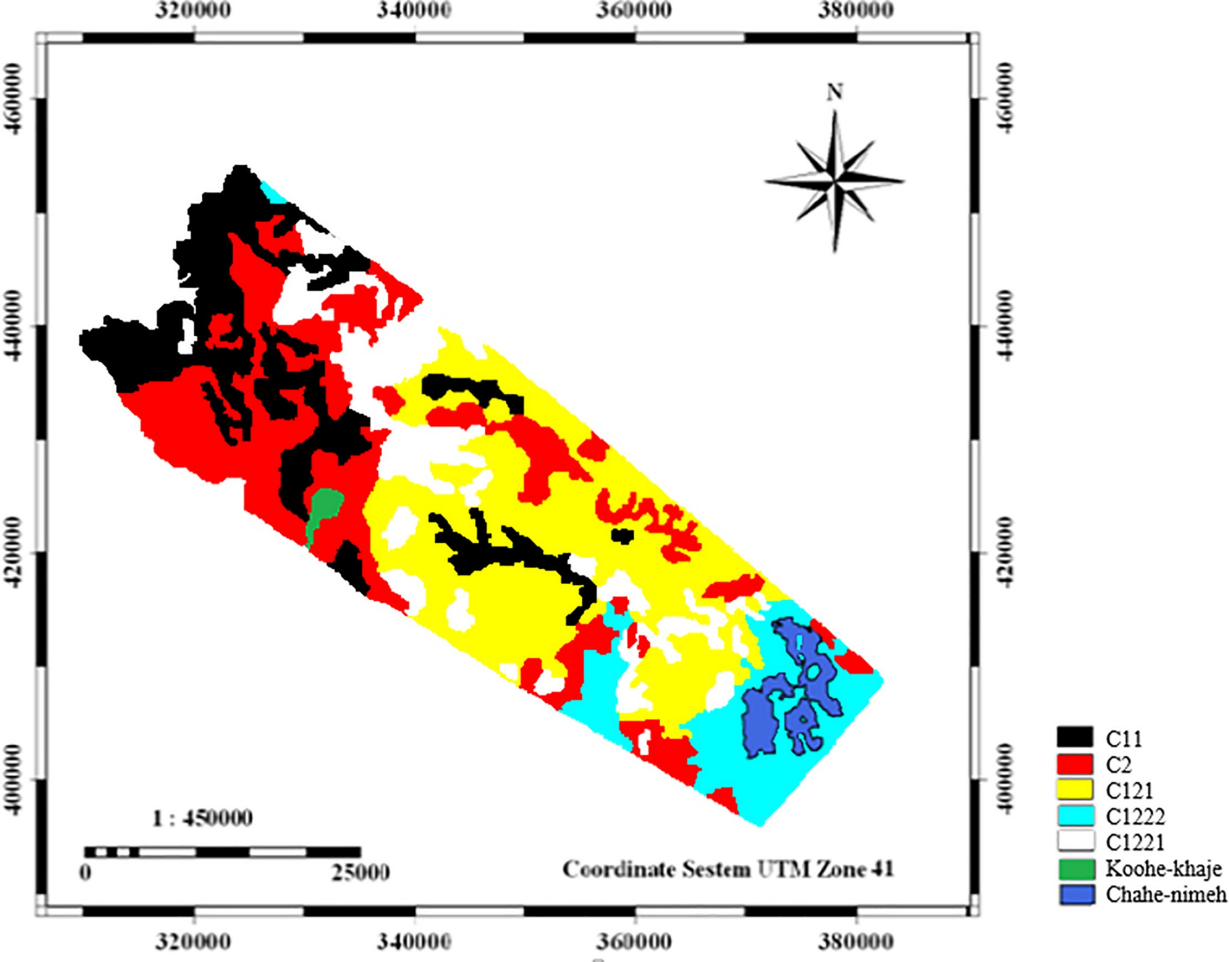
Map of work units based on cluster analysis for similar management decision (C11: facies 4,6,8,9,16; C2: facies 1,5,7,11,15; C121: facies 12,13; C1222; facies 2; C1221: facies 3,10,14,17).

Finally, the last cluster has five working units including fine sediments, salina, gravelly plain with the ups and downs and dry and hard shell and often *Artemisia scoparia*, *Tamarix aphylla*, *Zygophyllum eurypterum*, *Suaeda fruticosa* and *Atriplex canisens* with about 30 percent canopy are seen in these facies. In the C2 cluster, improving vegetation, seedling, and saplings in the favorite years and consideration to economic issues and livelihoods of villagers located near these regions are the management priorities. Furthermore, measures must be taken to prevent collecting branches in these areas. The results of cluster analysis showed different classification of working units or geomorphologic facies (Fig 14).

The prepared map of desertification intensity showed that all study units were classified in two classes of moderate and severe. However, field visits and evidence indicated that the work units located in the moderate or severe classes had significant differences. Regarding the scores given to the indicators and indices based on the desertification model, the results of cluster analysis showed that the initial work units studied fell into five different clusters. The units in each cluster have similarities in terms of all the indices studied.

**Fig 15:**
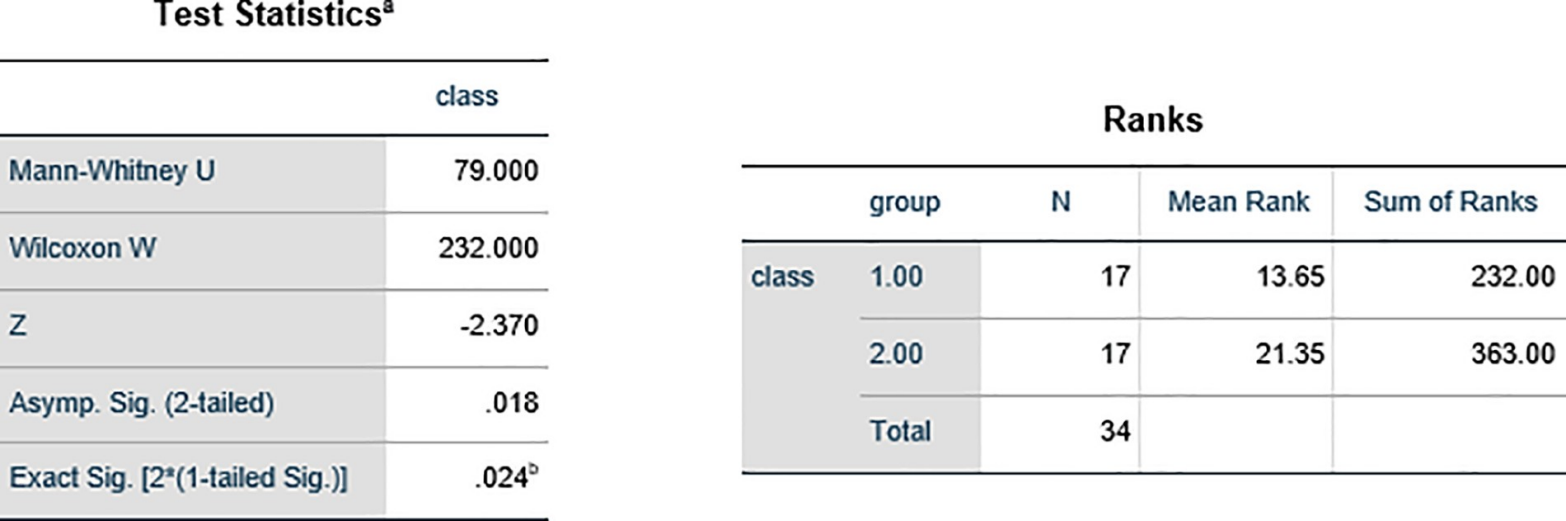
The uncertainty estimation of two methods (hierarchical cluster and desertification model) using Mann-Whitney test.

We used Mann-Whitney nonparametric test in spss23 software to assess the accuracy of the results of desertification model and clustering (Fig 15). The results of the Mann—Whitney test showed that the value of the test statistics was 79. Also, the value of Asymp.Sig was obtained to be 0.018, which is less than 0.025 (two-tailed test), and it can be concluded that the classification of work units in the two models, clustering and desertification, is not equal (P<0.05).

It is easy to see that although desertification intensity mapping is very common to identify the same intensity classes, performing field surveys and providing methods for managing the units located in the same class will encounter problems. Therefore, in addition to using desert intensity mapping models, it is recommended to use clustering method based on similarity recognition.

According to obtained results variations of MEDALUS classification and cluster in different working units depend on climate, soil, vegetation and wind erosion criteria, and their indicators.

The results of this classification can be used to determine geomorphological groups based on soil properties [52] and reduce the classification errors in planning and implementation of management projects in desert regions [53]. In this case, the implementation of desertification in working units of the same cluster can be done as the same. We can save the time, administrative and personnel costs with the same activities in homogenous units determined by cluster analysis. Therefore, it can be concluded that using cluster analysis has a good performance in grouping homogeneous environmental management units (HEMUs).

## Conclusions

In this study, the combination of a desertification mapping model and hierarchical analysis showed that different working units with the same desertification severity require different management decision. In most desertification severity mapping, the combination of different criteria is used, so the effective criterion in two similar work units may be different. Two working units (1 and 4), in this study, were categorized as medium severity of desertification but were subjected to different clusters (respectively C2 and C11) due to different affected criteria. Therefore, we suggest using cluster analysis to identify the same units, which need the same management decision after preparing the desertification intensity maps based on the conventional models.

The main basis of the proposed management plans in this study was the use of the UNEP ecosystem management program (UNEP 2004), with some modifications. According to the conditions of the study area and based on desertification intensity classes, some management plans, proportional to the conditions of each group of the same work units in the same cluster, were provided and recommended by experts (Table 7).

**Table 7:** The management plans recommended by experts for geobiofacies located in the same cluster.

Cluster Number	Facies Code	Proposed Management Plans
C11	4,6,8,9,16,18	the pebble mulch and spreading of vegetation residues are used on the surface to prevent the deterioration of surface erosion and maintenance of surface soil is the best and appropriate function.
C121	12,13	using biological windbreak and prevent livestock entry.
C1221	3,10,14	clay mulch.
C1222	2,17	restoring vegetation and using the exclusion of the area to give sufficient opportunity for plant regeneration.
C2	1,5,7,11,15	improving vegetation, seedling, and saplings in the favorite years and consideration to economic issues and livelihoods of villagers located near these regions are the management priorities
